# Examining an Alternate Care Pathway for Mental Health and Addiction Prehospital Emergencies in Ontario, Canada: A Critical Analysis

**DOI:** 10.3390/ijerph21020146

**Published:** 2024-01-29

**Authors:** Petra Meijer, Polly Ford-Jones, Dustin Carter, Patrina Duhaney, Simon Adam, Danielle Pomeroy, Sheryl Thompson

**Affiliations:** 1 Humber Institute of Technology & Advanced Learning, Toronto, ON M9W 5L7, Canada; polly.ford-jones@humber.ca (P.F.-J.); danielle.pomeroy@humber.ca (D.P.); sheryl.thompson@humber.ca (S.T.); 2 Middlesex–London Paramedic Service, London, ON N6E 1R4, Canada; dcarter@mlems.ca; 3 Faculty of Social Work, University of Calgary, Calgary, AB T2N 1N4, Canada; patrina.duhaney@ucalgary.ca; 4 School of Nursing, Faculty of Health, York University, Toronto, ON M3J 1P3, Canada; siadam@yorku.ca

**Keywords:** mental health emergency, alternate destination, social determinants of health

## Abstract

Paramedics in Ontario have largely been limited to transporting those with mental health or addiction (MHA)-related emergencies to the emergency department (ED). The ED has repeatedly been identified as a problematic and challenging setting for people with MHA needs. This article examines an innovative patient care model (PCM) established by the Middlesex–London Paramedic Service and its partners for specific MHA emergencies where patients were given options for care that included transportation to a Canadian Mental Health Association (CMHA) Crisis Centre or information for support. Qualitative and quantitative data that were utilized for regular reporting to the Ministry were included in the analysis. The findings indicated that the goals of reducing pressures on EDs and paramedic services, enhancing paramedics’ ability to address MHA calls, and improving patient care experiences were met. This model improves patient autonomy and options for care, improves the means for addressing patients’ social determinants of health, and offers transportation to a non-medicalized facility.

## 1. Introduction

Mental health and addiction-related emergency calls to 9-1-1 have been increasing in Ontario [1,2]. This is resulting in a shifting role for paramedics as they are increasingly responding to these mental health and addiction (MHA)-related emergencies [1]. A number of challenges impact paramedics’ ability to provide appropriate and responsive care to people experiencing crisis, including limited training and education with mental health needs [3,4,5] and limited on-scene resources or options for mental health support other than from paramedics [4,5].

Until 2019, under ministry regulation the emergency department (ED) was the only available transport destination for land ambulances [3,4,5]. The ED has been repeatedly identified as a problematic and challenging setting for those in crisis or with mental health needs [5,6,7,8]. Peng et al. [9] identified that a growing demand and limited resources in EDs have resulted in what they describe as ‘overcrowding’ where the demands of patients cannot be met within reasonable timeframes. Specifically, within the context of MHA-related needs, individuals with MHA needs are frequently found to be released from the ED without significant treatment, referral, or other next steps in care [4,10]. While EDs are appropriate for certain medical and trauma emergencies, they are not suitable for many, if not most, MHA-related needs [11]. Challenging conditions within the ED include long wait times; lack of privacy; lack of adequate supportive care beyond the presence of a security guard; and a generally chaotic, overstimulating environment [12]. Given these challenges, some paramedic services, along with existing community-based services, have identified a need for improved and alternative care options for this patient population. To allow paramedic services to implement new types of patient care models, the Ontario Ministry of Health (MoH) developed a patient care model (PCM) standard ‘to provide timely access to definitive care where options other than transport to the emergency department may be done safely and appropriately’ [13]. 

Paramedic services have been collaborating with several community-based mental health and addiction services such as the Canadian Mental Health Association and other local organizations to address both the long wait times in the ED and the needs of people with mental health and addiction emergencies. In Sudbury, for example, Sudbury Paramedic Services developed a ‘Mental Health and Addictions Triage and Transport Protocol’ in 2015 to allow paramedics to triage patients on scene for possible diversion from the ED [14]. Most recently, Toronto Paramedic Services has established a program to transport eligible patients to its ‘Stabilization and Connection Centre’ to deliver care to people experiencing homelessness who do not need acute medical care but are intoxicated by alcohol [15], and social workers and paramedics in Ottawa have teamed up to offer an alternative to the ED [16]. While the Association of Municipalities of Ontario has urged for shared learning from pilot projects on alternate models of care [17], there has been limited in-depth analysis of what makes these pilot projects work, how these projects impact patient care and paramedic practice, and how to improve the overall healthcare system for people dealing with acute mental health emergencies.

This article examines how the Middlesex–London Paramedic Service (MLPS), with local partners, has developed an alternate destination protocol for specific MHA emergencies with the goal of providing both higher-quality care for patients as well as reducing pressures on EDs. Specifically, we examine how the pilot impacted (1) ambulance visits and offload delays in the ED, (2) paramedics’ ability to manage MHA calls, and (3) patient care experiences for MHA-related emergencies. The history, development, and structure of the program are described, along with program impacts and challenges with implementing this alternate destination protocol. The need for care options that address the social determinants of health (SDOH) is discussed, as well as patient care models for MHA emergencies that enhance patient autonomy, options for care, and less medicalized approaches to care with an orientation toward recovery. The lens guiding this analysis is a ‘social determinants of health’ framework [18,19] informed by the critical mental health literature [20,21,22]. 

## 2. Materials and Methods

The MLPS Mental Health and Addiction Alternate Destination program was selected for this article because it was one of the first of its kind to be developed and has been one of the longest-running patient care models in Ontario, Canada. This program is the first formally established and approved program by the Ministry of Health in Ontario to provide a non-medicalized option for paramedics on the scene of an emergency mental health call to transport the patient directly to a non-designated community-based destination where patients can connect with mental health practitioners in the community. 

For this article, we used MLPS evaluation data from October 2017 to March 2022, encompassing Phase I and the first part of Phase II of the project. The MLPS compiled an evaluation report every four months for its PCM reporting to the Ontario Ministry of Health from March 2020 to March 2022, which included quantitative data from electronic patient care records and qualitative data from surveys to patients.

The qualitative reporting data included data from the ‘Ontario Perception of Care for Mental Health and Addictions’ survey completed by patients who were seen at the alternate destination site and a separate client satisfaction survey. The MLPS also compiled statistics from the electronic patient care records in the paramedic service iMedic database that included age distribution, gender distribution, Canadian Triage Acuity Scale (CTAS) levels, and ED disposition. Based on these data, the MLPS reported on the number of patients transported to the Canadian Mental Health Association (CMHA) Crisis Centre, patient outcomes, Crisis Response Team activation, the number of patients provided with Crisis Centre Reach-Out information, the number of eligible patients, patient’s primary problem and the patient or caregiver satisfaction score with the alternate destination. Data included in this manuscript were secondary data, and ethics approval was not required as data were obtained by the MLPS for program quality improvement. Quality improvement was described as an evaluation and review of the new patient care model intended to inform ongoing changes and the amelioration of the existing program. All data contained within the reports were deidentified.

Along with the evaluation reports prepared for the MoH, the authors analyzed the business cases of the project, reports of interviews with patients and paramedics, annual evaluations by the MLPS, reports by the MoH, news media, and reports from CMHA Middlesex. Data were also provided through the intake form completed by paramedics at the CMHA, CMHA patient outcome reports, and post-assessment follow-up. These data sources were each read through at least once and then systematically reviewed. These data were examined in relation to how the pilot goals were met, namely impacts on ambulance visits and offload delays to the ED, paramedics’ ability to manage MHA calls, and patient experiences. We also carried out a critical analysis of the nature of the program, informed by a ‘social determinants of health’ framework [18,19] and the critical mental health literature [20,21,22]. Critical analyses of health systems’ issues have been used to problematize dominant narratives and call attention to social determinants of health [1], and here, we challenge contemporary assumptions about practices and priorities in response to mental distress.

Phase I of the project reflected existing legislation (Ambulance Act 1990), and paramedics were only allowed to bring patients to destinations that were designated facilities under the Health Insurance Act (1990) and Public and Private Hospitals Acts (1990). This iteration utilized a Satellite CMHA Crisis Centre located within the hospital with data from October 2017 to February 2020. After the approval of the Patient Care Model Standards in 2019, the CMHA Crisis Centre moved to the CMHA Crisis Centre site in March 2020. All data included in this analysis were compiled as part of the initial pilot project and PCM reporting to the MoH and were provided by the MLPS.

Acknowledging the existing critique of the term ‘patient’ in many mental health contexts [23,24], this paper uses the term ‘patient’ conforming to the language used in paramedic services, referring to an individual for whom a request for an ambulance was made, and for whom a paramedic may provide assessment or care, regardless of transport by ambulance [25] (p. 8).

## 3. Results

### 3.1. History and Development of the Alternate Destination Pathway

#### 3.1.1. Program History and Objectives

The Middlesex–London Paramedic Service provides healthcare for a population of 515,000 in Southwestern Ontario. Healthcare changes in Ontario such as the deinstitutionalization of people with mental health needs and the closure of hospital psychiatric beds contributed to an increase in mental health emergencies [26,27]. In Ontario, starting in the 1960s, long-stay patients in large psychiatric institutions were reduced but without the associated funding or coordination of care for those in the community [28]. Currently, EDs and hospitals in Ontario continue to see an increase in the number of patients experiencing addictions and mental health-related emergencies [29]. In December 2015, CMHA Middlesex established a crisis walk-in clinic as part of the collaborative efforts of community and hospital services, individuals with lived experience, families, and the South West Local Health Integration Network (SWLHIN) with the goal of reducing barriers to crisis support. CMHA established and formalized a partnership and protocol with the London Police Service to facilitate the ‘warm transfer’ of individuals in mental health and/or addiction crisis from the police to the CMHA Crisis Centre. A ‘warm transfer’ indicates a transfer of care between care providers in front of, and with the patient, to provide them opportunity to clarify their perspective and be engaged in their care.

In 2017, the MLPS, SWLHIN, CMHA-Middlesex, and London Health Sciences Center (LHSC) began to work together to address the increased pressure on paramedic services and EDs caused by mental health emergencies. The objectives of this collaboration were to (1) reduce unnecessary ambulance visits to the ED, (2) reduce offload delays for paramedics, (3) enhance the ability of paramedics to manage mental health calls appropriately, and (4) enhance the individual and caregiver experience in dealing with mental health issues. The group looked at pilot projects in Sudbury [30] and Ottawa [31] for inspiration on how to structure the project. To achieve its objectives, the collaborative partners agreed to (1) establish a mental health crisis centre where paramedics could bring patients, (2) develop a protocol for identifying eligible ‘patients’ who had requested help from paramedics, and (3) a process for paramedics and patients to choose the most appropriate care.

#### 3.1.2. Development of the Satellite CMHA Crisis Centre

At the start of the collaboration, CMHA Middlesex already provided mental health crisis support through its Crisis Centre at 648 Huron Street in London. People experiencing a mental health crisis could walk into the Crisis Centre for immediate help, and no referrals were needed. Through collaboration with the London Police Service, clients with a mental health crisis could be transferred from the police to the Crisis Centre. Under the Ambulance Act (1990), however, paramedics in Ontario are required to transport patients to a designated receiving facility. In practice, this means that paramedics can transport patients to hospitals but not to any community-based facility. To comply with existing regulations, a Satellite Crisis Centre within the ED of LHSC Victoria was established in October 2017. The Satellite Crisis Centre was staffed by LHSC and CMHA Middlesex staff with close collaboration between partners. The Satellite Crisis Centre was able to provide the service on weekdays from 8 am to 2 pm due to limitations in funding and infrequent use of the centre during the startup phase. The project was continuously evaluated by the partners through weekly meetings and rapid cycle improvement rounds. During Phase II of the project, the CMHA Crisis Centre moved back to its site at 648 Huron Street and began to operate 24/7.

Concurrently, the MoH in Ontario began working on legislative changes under its Patient Care Model Standards for selected 9-1-1 patients to enable alternative destinations for paramedics. The MoH recognized the changing role of paramedics, and the pressure on the ED, and aimed for these initiatives ‘to divert patients away from emergency departments and reducing repeat hospital visits, which helps reduce patient wait times and ensures these hospital beds are available for those that need them most’ [32].

#### 3.1.3. Development of the Mental Health and Addiction Alternate Destination Protocol

In order to identify which 9-1-1 patients would be eligible to be offered to go to the CMHA Crisis Centre, the MLPS developed a Mental Health and Addiction Alternate Destination Protocol. Paramedics in Ontario provide emergency assessment, care, and transportation to the most appropriate care, based on standards. Paramedics in Ontario are regulated by the MoH and perform care based on the Basic Life Support Patient Care Standards (BLS PCS) and Advanced Life Support Patient Care Standards (ALS PCS) [33,34] under the direct authorization and control of a medical director of a regional base hospital. For example, paramedics are trained to recognize a cerebrovascular accident (stroke) through a specific set of assessments, identify possible contraindications, and then determine the best possible hospital for these patients, potentially bypassing the closest ED to go to a specialized hospital. Other destination guidelines (or bypass protocols) exist for situations, including, but not limited to, trauma patients and specific types of myocardial infarction (STEMI) (BLS 3.4).

**Figure 1:**
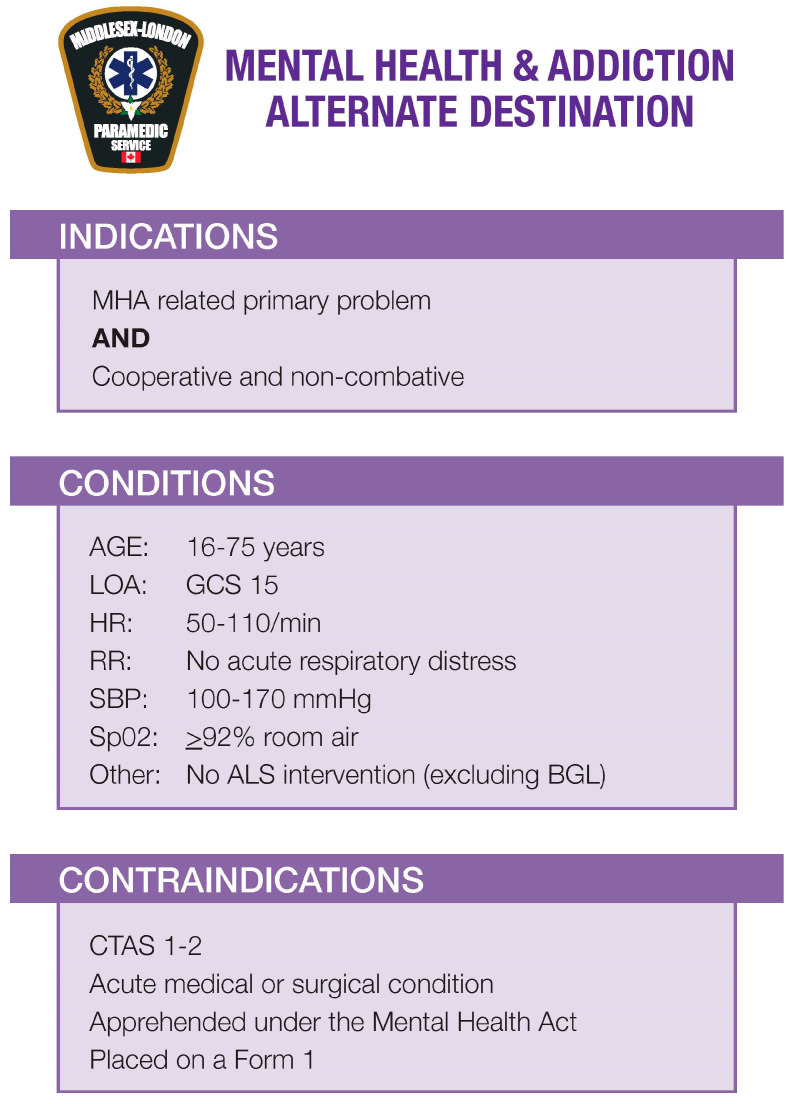

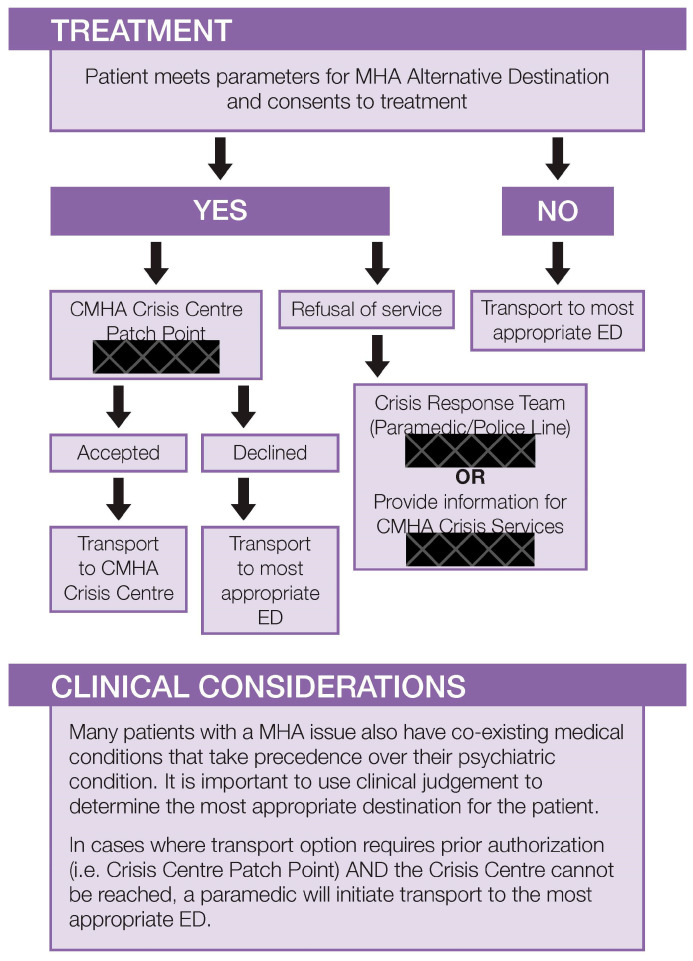
Mental Health and Addiction Alternate Destination Protocol (source: Mental Health and Addiction Alternate Destination Protocol Version 1.0. Middlesex–London Paramedic Service).

To develop its protocol, the MLPS assessed similar projects, including, among others, those developed in Sudbury and Ottawa. In 2014, Dr. Prpic and his team in Sudbury developed a ‘Mental Health and Addictions Triage and Transport Protocol’ that identified eligible patients based on a prehospital early warning score, patient presentation (which includes CTAS), and additional criteria [34]. The Sudbury program was the first approved trial pilot by the Emergency Health Services Branch of the MOH in Ontario to divert patients with mental health problems or intoxication to an alternate medical facility, bypassing the closest ED [14,35]. Based on the learnings of this program, the MLPS, in collaboration with the Medical Director of the South West Ontario Regional Base Hospital Program, LHSC, and CMHA London, developed the Mental Health and Addiction Alternate Destination Protocol for the MLPS (see Figure 1. Mental Health and Addiction Alternate Destination Protocol).

The Mental Health and Addiction Alternate Destination Protocol is structured based on the Medical Directives of the ALS PCS, including ‘indications’, ‘conditions’, ‘contraindications’, ‘treatment’, and ‘clinical considerations’. Paramedics in Ontario are familiar with this structure, and this was implemented to assist paramedics with utilizing the protocol. The conditions, indications, and contraindications were clearly defined and set at conservative values so that any existing or potential medical condition would take precedence over the mental health complaint. The ‘indications’ define the ‘general medical complaint or problem to which the Medical Directive applies’ [34], which in this protocol means any patient for whom their complaint was an MHA-related primary problem and when patients were cooperative and non-combative. The MLPS defined cooperative as patients who were able to provide consent for the alternate destination and able to engage in meaningful safety planning with the paramedics.

The conditions and contraindications define the clinical parameters that must be present for a treatment to be performed. Under the MHA Alternate Destination Protocol, only adult patients that can obey commands, are alert and oriented, have a normal heart rate (between 50 and 110 beats per minute), are normotensive (SBP between 100 and 170 mmHg), have no respiratory distress (with an SpO2 > 92%), and who have not received any ALS interventions would be eligible to be offered to go to the CMHA Crisis Centre. Patients would be contraindicated if they were CTAS 1 or 2, had an acute medical or surgical condition, were apprehended under the Mental Health Act, or were placed on Form 1.

The ‘treatment’ part of the protocol offers patients a choice to consent to receive transport to the CMHA Crisis Centre, refuse any further paramedic service, or be transported to the ED. The patient would be given information about the CMHA Crisis Centre so that the patient can make an informed decision about their next steps. Once a patient consented to be transported to the CMHA Crisis Centre, the paramedics would contact the CMHA Crisis Centre directly to see whether the centre would accept the patient. The patient would then either be transported to the CMHA Crisis Centre and transfer of care to the centre would take place, or, if the CMHA Crisis Centre declined, the patient would be transported to the most appropriate ED for transfer of care at the hospital. As with any 9-1-1 emergency, patients with capacity retain their right to decline any treatment or transport. In those cases, paramedics could provide information about CMHA Crisis Services or ask for consent to call CMHA’s Crisis Response Team to support the patient.

The ‘clinical considerations’ provide guidance to paramedics on the proper performance of a procedure or treatment. One of the greatest potential risks of the alternate destination is that the CMHA Crisis Centre is not a medical facility and does not have physicians on staff 24/7 for emergency crisis care. Paramedics must use their clinical judgement, including the wishes of the patient, to determine the best treatment options.

During the implementation of the project, paramedic staff at the MLPS were trained to use the Mental Health and Addiction Alternate Destination Protocol as part of their continuing medical education. Ontario paramedics are very familiar with the structure of a destination guideline and medical directive, so it was relatively straightforward, and with minimal cost, to implement the change. Once the Satellite Crisis Centre was open, paramedics were encouraged to offer the Mental Health and Addiction Alternate Destination Protocol to eligible patients. 

### 3.2. Findings

In 2020, the official MLPS Mental Health and Addiction Alternate Destination Patient Care Model was implemented, and after 4 months, the first formal evaluation of Phase I was submitted to the MoH. Evaluation reports were submitted every 4 months over the next 2 years as per the PCM Standards. The main purpose of the evaluations was to assess the patient safety parameters. The same data were also used to examine the effectiveness of the pilot project to reach its goals and understand whether the MLPS Alternate Destination Protocol could be effectively transitioned to a non-hospital location. The MLPS used the Quadruple Aim framework [36] as a framework for evaluation. Evaluation reports were submitted to the MoH after 12 and 24 months. In this section, we describe how this project impacted (1) ambulance visits and offload delays in the ED; (2) the enhancement of paramedics’ ability to manage MHA calls; and (3) patient care, including patient autonomy and the incorporation of social determinants of health for MHA emergency crises. 

#### 3.2.1. Reducing Pressure on the ED and Paramedic Service

**Figure 2:**
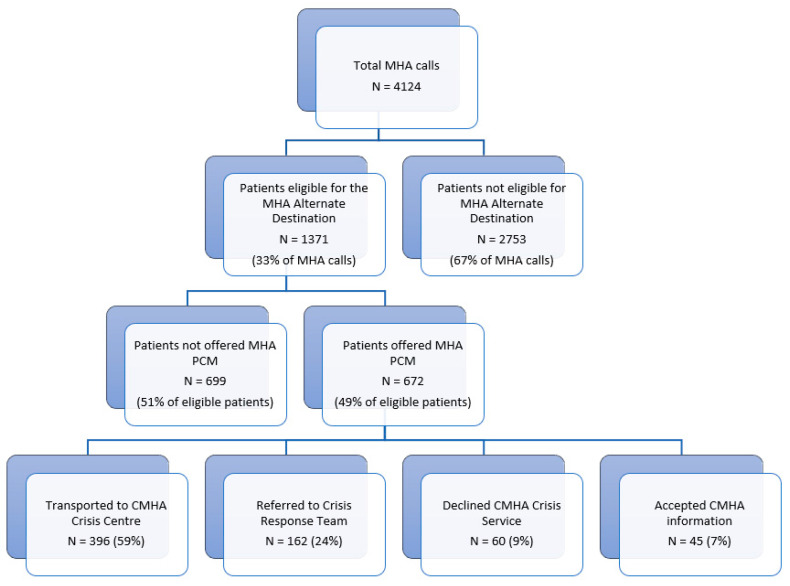
Visual representation of the results of MHA Alternate Destination Protocol uptake during the study period (from 2 March 2020 to 2 March 2022).

During the period between 2 March 2020 and 2 March 2022, a total of 4124 9-1-1 calls within the study criteria were recorded. Of those calls, 1371 (33.25%) patients met the conditions and indications of the Mental Health and Addiction Alternate Destination Protocol and were eligible to be offered transport to the Satellite CMHA Crisis Centre. Of these 1371 patients, 672 (49%) patients were offered an alternative destination, and of these 672 patients, 396 patients accepted transport (59%) (see Figure 2). While there were a few procedural challenges (mandatory patch point failure seven times where the paramedics could not reach the CMHA Crisis Centre, and 28 patients that were declined by the centre), a large majority of the patients were accepted by the CMHA Crisis Centre. During the first two years of the implementation of the project, between 66 and 115 patients per 4 months were directly transported to the CMHA Crisis Centre, reducing ambulance visits to the ED. Only a very small percentage of these patients (34 people over the two-year study period) presented to the ED within a predetermined time frame after visiting the CMHA Crisis Centre. Notably, 24% of patients treated at the Satellite Crisis Centre had been ‘frequent ED users’, with five or more ED visits in the month before they visited the CMHA Crisis Centre, indicating that providing patient care at the alternate destination may further reduce ED visits. Furthermore, around 7% of the patients who used the MHA Alternate Destination Protocol were transported to the alternate destination more than once per month. Of all the patients presented with the MHA Alternate Destination Protocol, 81% avoided going to the ED within a predefined timeframe as defined by the MOH evaluation framework after transport to the CMHA Crisis Centre.

Offload times for paramedics were reduced by approximately 50% for those patients who fit the criteria for the MLPS MHA Alternate Destination Protocol, allowing paramedics to get back on the road more quickly to respond to other calls in the community. The MLPS calculated that the average time for the transfer of care of a patient at the CMHA Crisis Centre was approximately 11 min. Based on the average offload delay in the hospital, the MLPS concluded that offload delay was reduced between 26 h (the period between 1 November 2021 and 2 March 2022) and 146 h (the period between 1 July 2020 and 31 October 2020) with an average of around 35 h per 4 months, with an estimated average cost savings of CAD 45,000 per 4 months.

#### 3.2.2. Enhancing Paramedics’ Ability to Manage Mental Health and Addiction Emergency Calls

The MLPS conducted a survey in February 2021 to understand the provider experience with the protocol, to which 71 out of 190 participants responded. The vast majority (96%) of the paramedic respondents rated their experience using the MHA Alternate Destination Protocol as a 4/5 or 5/5 in terms of satisfaction with the protocol. The same percentage stated that they understood the protocol/procedure. The paramedics overwhelmingly supported the protocol and the partnership with the CMHA and indicated that this protocol enhanced patient care. As one paramedic commented in the survey comments:

‘This is a wonderful service that puts patient care first and is EXTREMELY beneficial for patients that meet the protocol. I hope this protocol sticks around for good!’

Another paramedic also commented on the protocol’s ability to place patient care and needs first and that it better serves mental health patients compared to the ED.

‘It would be uncommon for someone to go to the ED and have an hour-long crisis intervention and get the appropriate services they need. The ED isn’t set up for mental health patients—it’s very rushed and they have to talk quickly about what’s going on’.

One of the challenges identified with the implementation is that over 50% of the eligible patients were not offered the MHA Alternate Destination Protocol. The survey revealed that about a third of paramedics stated that they would sometimes forget to offer the option of an alternate care pathway to prospective patients. Some paramedics thought that a checklist or regular refresher would help to better implement the protocol. As one respondent stated:

‘Refreshers would be awesome, having the prompt card handy is the best tool. I don’t use it enough to know everything off the top of my head’,

and another added that

‘It would be best to have a reminder of the fast link for access on cell phone or integration in the Base Hospital App’.

Paramedics were provided annual retraining updates, patient outcome reports, and other reminders to support the utilization of the protocol. There were no reported adverse medical events associated with the protocol demonstrating success with the protocol and training provided to paramedics. The paramedics and CMHA staff spoke positively about the collaboration, citing that they learned a lot from the interaction and felt better prepared to respond to addictions and mental health-related calls. 

#### 3.2.3. Improved Patient Care and Experience

The results of the patient surveys showed that patients largely had positive experiences at the CMHA Crisis Centre and stated that their needs were met. Many patients indicated that the centre helped them significantly and provided support. Patients reported that they liked having the choice to go to the CMHA Crisis Centre. Participants commented:

‘Paramedics explained it well. I didn’t know going to the CC was an option before and was happy for it. It was “calmer” than going to the ED. I have referred CC and Reach Out to other friends. It’s awesome that you don’t have to wait for six hours’.

‘Having the option to come to the CC was a huge relief—did not want to go to the ED’.

The CMHA Crisis Centre provides ‘assessment and supportive counseling for immediate crisis issues and referrals to other services for ongoing non-crisis issues’ [37] (CMHA Thames Valley). The CMHA Crisis Centre does not have prescription medication or provide psychiatric consults. It is important for patients to provide informed consent on their choice of care, and paramedics should explain the options. A large majority (over 80%) of the patients who responded to the surveys indicated that the paramedics explained the services of the Crisis Centre, which allowed them to make a well-informed decision. Some, however, indicated that they did not fully understand the options as explained by the paramedics or that the paramedics could not explain what services the CMHA Crisis Centre would provide for them. Otherwise, patients commented positively on the additional services that the CMHA provided compared to the ED. The CMHA offers regular follow-ups with patients and can provide patients with different options after the initial crisis has been settled, such as connection to housing services, addiction support, or help with family issues. 

#### 3.2.4. Challenges with the Implementation

One of the major challenges with the implementation of the protocol from the provider’s perspective was that about 50% of eligible patients were not offered or transported to the Satellite Crisis Centre. MLPS conducted research to better understand which potentially eligible patients were offered the MHA Alternate Destination Protocol and which were not offered the protocol. Emergencies that were identified as ‘situational crisis’ had the highest percentage of acceptance, while ‘auditory disturbance’ and ‘emotional distress’ had the lowest acceptance. We identified five main reasons for not offering the MHA Alternate Destination Protocol to eligible patients. These included paramedics forgetting to offer the protocol (33%), being uncertain about patient eligibility (34%), lack of training/knowledge on mental health (8%), lack of training/knowledge on MHA Alternate Destination Protocol (8%), and other (17%). Although paramedics generally had a positive experience, some paramedics found that the transfer between paramedic care and the CMHA Crisis Centre care was not always straightforward.

‘I do find the staff has been very reluctant to accept the patients that I have referred to them even though they meet the protocol that we have at our disposal. The staff don’t seem comfortable with the description of the patient conditions we give over the phone. I found the same thing with the CC when we were alternately transporting patients to them at LHSC. With the exception of that it is generally a seamless experience’.

One of the reasons for some of the challenges in patient transfer is that paramedics and CMHA Crisis Centre staff have different descriptions for patient presentation, and interfacility transfer of patients can be difficult [38]. The strong collaboration between the MLPS, CMHA, and the hospital on this PCM assisted in supporting the transition between paramedics and the CMHA Crisis Centre. 

While the CMHA Crisis Centre could provide services to patients that the ED could not provide, not all eligible patients wanted to go to the CMHA Crisis Centre due to its location away from the inner city (~5 km from the inner city), a lack of understanding of the services provided, and the lack of a physician at the centre to prescribe medication. These patients either wanted to be transported to the ED (which was an option offered to them) or refused any further support from paramedics. 

## 4. Discussion

Significant challenges have been identified with the current approaches to emergency mental health response [5,39]. Interactions with those in crisis have the potential to significantly impact the level of distress and de-escalation, as well as future trust and interactions with mental health services [7]. While 9-1-1 calls for mental health-related needs have been often deemed ‘inappropriate’ or ‘misuse’ of paramedic services and ED services [40,41], individuals are often forced to turn to emergency services when no other options exist [1,42]. In many cases, there is a need to connect patients to more appropriate care options to address the needs that have driven them to seek paramedic care to begin with [43]. A critical mental health lens offers an approach of systematic critical thinking, considering practices, priorities, and knowledge bases in the areas of mental health [20]. This offers a means of critically examining existing mental healthcare options and approaches and considering what might better serve those in distress, not taking for granted existing paradigms.

Historically, and to this day, mental health has been understood with a reductionist, biomedical approach, placing emphasis on the biological, neurotransmitters, and genetics [44] and considering people’s distress as individualized, one that requires medical or health system interventions [21,22]. More critical approaches to understanding mental health have highlighted the importance of context, including personal histories, relationships, cultural and political systems, and the policies that result [21,45]. Following this rationale, more critical understandings of mental health view people’s distress and crisis as significantly impacted by complex, immensely challenging living conditions, and social injustices [21,46], including the social determinants of health [18,19,47,48,49]. The social determinants of health refer to the ‘quantity and quality of a variety of resources that a society makes available to its members’ [50] (p. 2) and acknowledge that factors such as income, employment, housing, working conditions, food security, social isolation, racialization, and gender, among others, intersect and significantly impact people’s mental health and wellbeing [17,48,50]. Incorporating these contexts in understanding distress, offering non-pharmacological options for intervention and support, and providing supportive care options not based in hospital settings can be considered non-medicalized approaches to care. In looking to comprehensively and appropriately meet patient needs when experiencing a mental health-related emergency, it is necessary then to consider non-medicalized approaches to support and care.

### 4.1. The Alternate Destination Protocol as a Non-Medicalized Approach to Mental Health Emergency Care

The MLPS Alternate Destination Protocol importantly offers an alternative to hospital, ED-based transport, and care. Firstly, offering a non-medical care destination, outside of a hospital, with non-medical, supportive mental healthcare professionals is an important step in a de-medicalized approach to mental health and mental healthcare, where the ED has been repeatedly identified as a problematic destination for those with mental health specific needs or crisis [2,3,4]. This is a significant first step in a system where multiple policies and structures, such as the Ambulance Act (1990), the Public Hospitals Act (1990), and the Ontario Health Insurance Act (1990) have previously prohibited the possibility of an alternative destination such as this or made it exceptionally challenging. Secondly, this program involves enhancing patient autonomy, where the person in need of care can decide what will best suit their care needs. Throughout history and to this day, the mental health system has significantly or at times entirely restricted people’s agency, denying them the ability of self-determination, despite the significant role of personal agency in moving toward recovery [51]. Those with mental health needs or those who are in crisis are often presumed to be not rational and without knowledge of what is best for themselves [51]. Many approaches to mental healthcare utilize significant power imbalance and coercion, where those experiencing distress have little or no say in their care [52]. In offering patients a choice between transport to the ED and an alternate destination, patients’ autonomy is increased, and they are also offered the possibility of transport to a non-medical destination, which may well provide a setting with more choices about the type of care they receive. Importantly, the protocol also continues to provide those in distress with the option of receiving hospital-based care, in the ED if they so choose. While there are still limitations to individuals’ choices and autonomy in this context, in terms of the options (decline transport, transport to the ED, and now transport to an alternate destination), it is an important improvement. 

In terms of patient experience, quality of care, and follow-up, the ability of the alternate destination site to provide care extending beyond medical care offers the potential to meet patients’ social and material needs. Such follow-up and connection to more appropriate resources may not only address the particular emergency that led the person to encounter paramedic services but may actually prevent future crises. While engagement at structural and policy levels beyond the healthcare system is crucial for addressing and improving individuals’ SDOH, interventions that work to address social determinants can significantly improve mental health outcomes [53,54]. Paramedics are identified as having ‘untapped’ potential to address the SDOH [55], and this alternate destination program offers a significant opportunity for paramedics and paramedic services to address SDOH-related needs at a point of crisis or urgent need. By connecting those in distress with resources to support their material needs and social determinants, people’s distress may, at least to some degree, retain context and meaning.

### 4.2. Implications for Paramedic Services

The findings from this study have implications for paramedic services in Ontario. The MLPS and their collaborating organizations identified goals of both reducing pressure on EDs and improving the quality of care for those experiencing MHA needs. As paramedic services [1,2] and EDs [11] face substantial and increasing pressures in Ontario, doubtless, the goal of reducing these pressures on both services is significant. As identified by the experience of the MLPS Mental Health and Addiction Alternate Destination Protocol, offering an alternative care destination other than ED services improved these pressures and associated costs. Crucially, with a goal of improving care experiences and options for those experiencing mental health and addiction-related emergencies, this program offered improvement to patients’ autonomy, their options for care, and a non-medicalized approach that centred more around supportive care and means of addressing patients’ relevant SDOHs. 

As paramedic services consider means of addressing both goals and, specifically, better meeting the needs of patient populations with mental health and addiction-related emergencies, an alternative care pathway offers a promising and, to date, safe option. Partnership to meet the needs and goals of both community-based mental health organizations and their clients, as well as paramedic services, presents a crucial point of collaboration for addressing this need. While community partners and relevant geographical contexts vary significantly among Ontario communities, the potential for alternate care options and some iteration based on lessons learned from the MLPS program offer an important direction for change and implementation for other paramedic services. 

This paper has some limitations. Firstly, it specifically involves the MLPS, CMHA Middlesex, and other geographically relevant partner contexts. The specific population demographics, geographies, local, available social care services, and resources doubtless impact specific implementation possibilities and feasibility for care options. Another limitation of this paper is that it relies on secondary data, and participant interviews and evaluations were conducted by others outside of the authors of this paper. Finally, as program implementation and data collection occurred during the COVID-19 pandemic, increased pressures on EDs, paramedic services, and social services were exacerbated. Prior existing health inequities and mental health needs were exacerbated during the pandemic [56,57], and an ongoing assessment of program impacts will be warranted to investigate changes at the later stages of the pandemic and post-pandemic.

## 5. Conclusions

This article explores a model of care established by a paramedic service in collaboration with local, invested partners, working to address both system demands and enhanced support for those experiencing mental health and addiction-related needs. Based on program evaluation reports, the benefits of the program included improved efficiency for paramedic services; cost savings in offload delay times; reduced wait times for paramedics and patients alike; an improved care option, particularly for ‘frequent’ users of ED services; improved paramedic experience in providing care; and improved patient use satisfaction. While the program experienced growing pains related to protocol familiarity and the clarity of the program being offered to users, it has offered an innovative and positive care pathway for this patient population, and to date, the program has had no adverse outcomes, as measured by a subsequent medical emergency requiring transport to an ED and EMS re-accessing a patient within 72 h. The program has achieved the stated goals of a decrease in ambulance visits and offload delays of MHA-related emergencies, improved paramedic capacity to respond to MHA-related emergency calls with additional care options and clear care pathways, and positive reports of patient care experiences with the program.

Future research could explore the implementation of such a program within another geographical and service context, and further in-depth exploration of possible services offered in a crisis centre context is also required. Also warranted is further study of implications for particular demographics and populations, assessing the ways in which such a program meets the needs of diverse patient populations. 

The program, developed within existing system constraints and legislative barriers, has offered a mental healthcare option that enhances patients’ choice and autonomy in their care, non-medicalized options for care, and a point of connection to address patients’ SDOH-related needs. This model of care represents an important progression in the type of care and services paramedics may provide to those with mental health and addiction-related needs and a positive model for other paramedic services considering how to meet both service and patient needs.

## Data Availability

The data are not publicly available.
